# 3-(2,3-Dimethyl-5-oxo-1-phenyl-2,5-di­hydro-1*H*-pyrazol-4-yl)sydnone

**DOI:** 10.1107/S1600536810015667

**Published:** 2010-05-08

**Authors:** Jia Hao Goh, Hoong-Kun Fun, Nithinchandra, B. Kalluraya

**Affiliations:** a X-ray Crystallography Unit, School of Physics, Universiti Sains Malaysia, 11800 USM, Penang, Malaysia; b Department of Studies in Chemistry, Mangalore University, Mangalagangotri, Mangalore 574 199, India

## Abstract

In the title sydnone compound [systematic name: 3-(2,3-dimethyl-5-oxo-1-phenyl-2,5-dihydro-1*H*-pyrazol-4-yl)-1,2,3-oxadiazol-3-ium-5-olate], C_13_H_12_N_4_O_3_, the oxadiazole and pyrazole rings are essentially planar [maximum deviations = 0.006 (1) and 0.019 (1) Å, respectively] and are inclined at inter­planar angles of 37.84 (4) and 46.60 (4)°, respectively, with respect to the benzene ring. In the crystal, adjacent mol­ecules are inter­connected into a three-dimensional supra­molecular network *via* inter­molecular C—H⋯O hydrogen bonds. Weak inter­molecular π–π aromatic stacking inter­actions [centroid–centroid distance = 3.5251 (5) Å] further stabilize the crystal packing.

## Related literature

For general background to and applications of sydnone derivatives, see: Baker *et al.* (1949 ▶); Hedge *et al.* (2008 ▶); Rai *et al.* (2008 ▶). For the preparation of 3-aryl sydnones, see Kalluraya *et al.* (2004 ▶); Rai *et al.* (2008 ▶). For related structures, see: Baker & Ollis (1957 ▶); Goh *et al.* (2009**a* ▶,b* ▶, 2010**a* ▶,b* ▶). For bond-length data, see: Allen *et al.* (1987 ▶). For the stability of the temperature controller used for the data collection, see: Cosier & Glazer (1986 ▶).
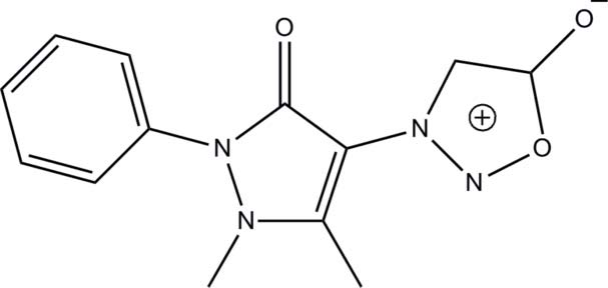


## Experimental

### 

#### Crystal data

C_13_H_12_N_4_O_3_
*M* *_r_* = 272.27
Monoclinic, 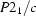

*a* = 10.6525 (3) Å
*b* = 7.3014 (3) Å
*c* = 15.6828 (4) Å
β = 93.982 (1)°
*V* = 1216.83 (7) Å^3^
*Z* = 4
Mo *K*α radiation
μ = 0.11 mm^−1^
*T* = 100 K
0.56 × 0.17 × 0.08 mm

#### Data collection

Bruker APEXII DUO CCD area-detector diffractometer
Absorption correction: multi-scan (*SADABS*; Bruker, 2009 ▶) *T*_min_ = 0.941, *T*_max_ = 0.992
44010 measured reflections
6426 independent reflections
4895 reflections with *I* > 2σ(*I*)
*R*_int_ = 0.048

#### Refinement

*R*[*F* ^2^ > 2σ(*F* ^2^)] = 0.042
*wR*(*F* ^2^) = 0.125
*S* = 1.04
6426 reflections
229 parameters
All H-atom parameters refined
Δρ_max_ = 0.55 e Å^−3^
Δρ_min_ = −0.41 e Å^−3^

### 

Data collection: *APEX2* (Bruker, 2009 ▶); cell refinement: *SAINT* (Bruker, 2009 ▶); data reduction: *SAINT*; program(s) used to solve structure: *SHELXTL* (Sheldrick, 2008 ▶); program(s) used to refine structure: *SHELXTL*; molecular graphics: *SHELXTL*; software used to prepare material for publication: *SHELXTL* and *PLATON* (Spek, 2009 ▶).

## Supplementary Material

Crystal structure: contains datablocks global, I. DOI: 10.1107/S1600536810015667/sj2767sup1.cif

Structure factors: contains datablocks I. DOI: 10.1107/S1600536810015667/sj2767Isup2.hkl

Additional supplementary materials:  crystallographic information; 3D view; checkCIF report

## Figures and Tables

**Table 1:** Hydrogen-bond geometry (Å, °)

*D*—H⋯*A*	*D*—H	H⋯*A*	*D*⋯*A*	*D*—H⋯*A*
C1—H1*A*⋯O3^i^	0.981 (14)	2.492 (13)	3.2155 (10)	130.4 (10)
C11—H11*A*⋯O2^i^	0.944 (13)	2.543 (13)	3.4163 (10)	153.9 (11)
C12—H12*B*⋯O2^ii^	0.984 (13)	2.369 (13)	3.3022 (10)	158.1 (11)
C13—H13*A*⋯O3^iii^	0.958 (14)	2.516 (15)	3.3606 (10)	147.0 (12)

Symmetry codes: (i) 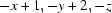; (ii) 
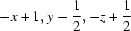
; (iii) 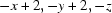.

**Fig. 1.**
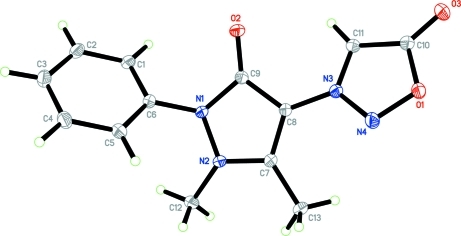
The molecular structure of the title compound, showing 50% probability displacement ellipsoids for non-H atoms and the atom-numbering scheme.

**Fig. 2.**
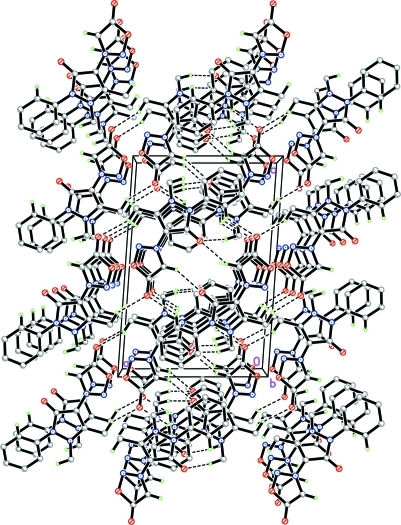
The crystal packing of the title compound, viewed along the b axis, showing a three-dimensional supramolecular network. Hydrogen atoms not involved in intermolecular interactions (dashed lines) have been omitted for clarity.

## supplementary crystallographic information

### Comment

Sydnones constitute a well-defined class of mesoionic compounds that contain a 1,2,3-oxadiazole ring system.  The introduction of the concept of mesoionic structure for certain heterocyclic compounds in the year 1949 has proved to be fruitful development in heterocyclic chemistry (Baker *et al.*, 1949). The study of sydnones still remains a field of interest because of their electronic structure and also because of the various types of biological activities displayed by some of them. Interest in sydnone derivatives has also been encouraged by the discovery that they exhibit various pharmacological activities (Hedge *et al.*, 2008; Rai *et al.*, 2008).

3-aryl sydnones are prepared by the cyclisation of *N*-nitroso-*N*-aryl glycines with acetic anhydride. These *N*-nitroso-*N*-aryl glycines were obtained by the nitrosation of *N*-substituted glycines. The *N*-substituted glycine was obtained by the hydrolysis of corresponding ester with sodiumhydroxide. The ester is in turn obtained by the reaction of appropriately substituted aniline with ethyl aceto acetate in absolute ethanol medium employing anhydrous sodium acetate as the catalyst (Kalluraya *et al.*, 2004; Rai *et al.*, 2008)

In the title sydnone compound (Fig. 1), the 1,2,3-oxadiazole (N3/N4/O1/C10/C11) and pyrazole (N1/N2/C7-C9) rings are essentially planar, with maximum deviations of 0.006 (1) and -0.019 (1) Å, respectively, at atoms N4 and N1. These two rings are inclined at interplanar angles of 37.84 (4) and 46.60 (4)°, respectively, with the C1-C6 benzene ring. As reported previously (Goh *et al.*, 2010*a,b*), the exocyclic C10–O3 bond length of 1.2205 (9) Å is inconsistent with the formulation of Baker & Ollis (1957), which involves the delocalization of a positive charge in the 1,2,3-oxadiazole ring, and a negative charge in the exocyclic oxygen. The bond lengths (Allen *et al.*, 1987) and angles are within normal range and comparable to those observed in closely related pyrazole (Goh *et al.*, 2009*a,b*) and sydnone (Goh *et al.*, 2010*a,b*) structures.

In the crystal packing, C1—H1A···O3, C13—H13A···O3, C11—H11A···O2 and C12—H12B···O2 hydrogen bonds (Table 1) form two different pairs of intermolecular bifurcated acceptor hydrogen bonds, which link the molecules into a three-dimensional supramolecular network. The crystal packing is further stabilized by weak intermolecular π–π aromatic stacking interactions [*Cg*1···*Cg*2^ii^ = 3.5251 (5) Å, (ii) = -x+1, y-1/2, -z+1/2 where *Cg*1 and *Cg*2 are centroids of pyrazole and benzene rings, respectively].

### Experimental

[(1,5-Dimethyl-3-oxo-2-phenyl-2,3-dihydro-1*H*-pyrazol-4-yl)(nitroso) amino]acetic acid (0.1 mol) was heated with acetic anhydride (0.5 mol) on a water bath for 2–4 h, the reaction mixture was kept aside at room temperature for overnight. It was then poured into ice cold water, filtered and washed with water, sodium bicarbonate solution (5 %) and again with water. The solid product was dried and crystallized from benzene. Single crystals suitable for X-ray analysis were obtained from a 1:2 mixture of DMF and ethanol by slow evaporation.

### Refinement

All hydrogen atoms were located from difference Fourier map [range of C—H = 0.944 (13)–0.993 (16) Å] and allowed to refine freely.

### Crystal data

**Table:**

C_13_H_12_N_4_O_3_	*F*(000) = 568
*M**_r_* = 272.27	*D*_x_ = 1.486 Mg m^−^^3^
Monoclinic, *P*2_1_/*c*	Mo *K*α radiation, λ = 0.71073 Å
Hall symbol: -P 2ybc	Cell parameters from 9368 reflections
*a* = 10.6525 (3) Å	θ = 3.1–37.4°
*b* = 7.3014 (3) Å	µ = 0.11 mm^−^^1^
*c* = 15.6828 (4) Å	*T* = 100 K
β = 93.982 (1)°	Block, brown
*V* = 1216.83 (7) Å^3^	0.56 × 0.17 × 0.08 mm
*Z* = 4	

### Data collection

**Table:**

Bruker APEXII DUO CCD area-detector diffractometer	6426 independent reflections
Radiation source: fine-focus sealed tube	4895 reflections with *I* > 2σ(*I*)
graphite	*R*_int_ = 0.048
φ and ω scans	θ_max_ = 37.6°, θ_min_ = 1.9°
Absorption correction: multi-scan (*SADABS*; Bruker, 2009)	*h* = −18→18
*T*_min_ = 0.941, *T*_max_ = 0.992	*k* = −12→12
44010 measured reflections	*l* = −26→26

### Refinement

**Table:**

Refinement on *F*^2^	Primary atom site location: structure-invariant direct methods
Least-squares matrix: full	Secondary atom site location: difference Fourier map
*R*[*F*^2^ > 2σ(*F*^2^)] = 0.042	Hydrogen site location: inferred from neighbouring sites
*wR*(*F*^2^) = 0.125	All H-atom parameters refined
*S* = 1.04	*w* = 1/[σ^2^(*F*_o_^2^) + (0.0663*P*)^2^ + 0.1919*P*] where *P* = (*F*_o_^2^ + 2*F*_c_^2^)/3
6426 reflections	(Δ/σ)_max_ = 0.001
229 parameters	Δρ_max_ = 0.55 e Å^−^^3^
0 restraints	Δρ_min_ = −0.41 e Å^−^^3^

### Special details

**Table:**

Experimental. The crystal was placed in the cold stream of an Oxford Cryosystems Cobra open-flow nitrogen cryostat (Cosier & Glazer, 1986) operating at 100.0 (1)K.
Geometry. All esds (except the esd in the dihedral angle between two l.s. planes) are estimated using the full covariance matrix. The cell esds are taken into account individually in the estimation of esds in distances, angles and torsion angles; correlations between esds in cell parameters are only used when they are defined by crystal symmetry. An approximate (isotropic) treatment of cell esds is used for estimating esds involving l.s. planes.
Refinement. Refinement of F^2^ against ALL reflections. The weighted R-factor wR and goodness of fit S are based on F^2^, conventional R-factors R are based on F, with F set to zero for negative F^2^. The threshold expression of F^2^ > 2sigma(F^2^) is used only for calculating R-factors(gt) etc. and is not relevant to the choice of reflections for refinement. R-factors based on F^2^ are statistically about twice as large as those based on F, and R- factors based on ALL data will be even larger.

### Fractional atomic coordinates and isotropic or equivalent isotropic displacement parameters (Å^2^)

**Table:**

	*x*	*y*	*z*	*U*_iso_*/*U*_eq_	
O1	0.93586 (5)	1.20867 (9)	0.00540 (4)	0.02015 (12)	
O2	0.51808 (5)	1.07724 (9)	0.11293 (4)	0.01697 (11)	
O3	0.83681 (6)	1.19975 (11)	−0.12824 (4)	0.02477 (14)	
N1	0.59770 (5)	0.96695 (9)	0.24595 (4)	0.01428 (11)	
N2	0.71755 (6)	0.94159 (9)	0.28563 (4)	0.01458 (11)	
N3	0.79599 (6)	1.09624 (9)	0.08032 (4)	0.01374 (11)	
N4	0.91049 (6)	1.16505 (11)	0.08800 (4)	0.01949 (13)	
C1	0.37495 (7)	0.95237 (11)	0.26642 (5)	0.01608 (13)	
C2	0.27426 (7)	0.99016 (12)	0.31562 (6)	0.01978 (14)	
C3	0.29319 (8)	1.07726 (13)	0.39442 (6)	0.02155 (15)	
C4	0.41384 (8)	1.12975 (12)	0.42404 (5)	0.01955 (14)	
C5	0.51539 (7)	1.09504 (11)	0.37507 (5)	0.01622 (13)	
C6	0.49514 (6)	1.00508 (10)	0.29691 (4)	0.01404 (12)	
C7	0.80407 (6)	0.97734 (10)	0.22937 (5)	0.01404 (12)	
C8	0.74125 (6)	1.03529 (10)	0.15426 (4)	0.01342 (12)	
C9	0.60776 (6)	1.03268 (10)	0.16264 (4)	0.01363 (12)	
C10	0.83202 (7)	1.16748 (12)	−0.05217 (5)	0.01728 (13)	
C11	0.74198 (7)	1.09301 (11)	−0.00008 (5)	0.01608 (13)	
C12	0.73637 (7)	0.82345 (12)	0.36075 (5)	0.01818 (14)	
C13	0.93987 (7)	0.94884 (12)	0.25274 (5)	0.01862 (14)	
H1A	0.3617 (12)	0.8870 (19)	0.2119 (9)	0.023 (3)*	
H2A	0.1903 (13)	0.953 (2)	0.2965 (9)	0.030 (3)*	
H3A	0.2226 (13)	1.100 (2)	0.4312 (9)	0.031 (3)*	
H4A	0.4263 (14)	1.193 (2)	0.4800 (10)	0.035 (4)*	
H5A	0.5999 (12)	1.134 (2)	0.3956 (8)	0.026 (3)*	
H11A	0.6593 (12)	1.0502 (18)	−0.0133 (9)	0.023 (3)*	
H13A	0.9830 (14)	0.939 (2)	0.2013 (9)	0.033 (4)*	
H13B	0.9704 (14)	1.055 (2)	0.2880 (10)	0.040 (4)*	
H13C	0.9524 (14)	0.841 (2)	0.2879 (10)	0.036 (4)*	
H12A	0.7723 (15)	0.894 (2)	0.4061 (10)	0.040 (4)*	
H12B	0.6549 (12)	0.7786 (18)	0.3781 (8)	0.024 (3)*	
H12C	0.7870 (14)	0.718 (2)	0.3489 (9)	0.033 (4)*	

### Atomic displacement parameters (Å^2^)

**Table:**

	*U*^11^	*U*^22^	*U*^33^	*U*^12^	*U*^13^	*U*^23^
O1	0.0160 (2)	0.0304 (3)	0.0140 (2)	−0.0050 (2)	0.00113 (18)	0.0030 (2)
O2	0.0129 (2)	0.0238 (3)	0.0137 (2)	0.00159 (19)	−0.00223 (17)	−0.00001 (19)
O3	0.0216 (3)	0.0389 (4)	0.0139 (2)	−0.0011 (2)	0.0019 (2)	0.0053 (2)
N1	0.0093 (2)	0.0214 (3)	0.0119 (2)	0.00001 (19)	−0.00034 (18)	0.0004 (2)
N2	0.0103 (2)	0.0204 (3)	0.0128 (2)	0.00010 (19)	−0.00083 (18)	0.0023 (2)
N3	0.0120 (2)	0.0165 (3)	0.0126 (2)	−0.00043 (19)	0.00062 (18)	−0.00050 (19)
N4	0.0159 (3)	0.0288 (4)	0.0138 (3)	−0.0064 (2)	0.0007 (2)	0.0014 (2)
C1	0.0118 (3)	0.0194 (3)	0.0169 (3)	0.0000 (2)	0.0002 (2)	0.0015 (2)
C2	0.0124 (3)	0.0245 (4)	0.0227 (3)	0.0014 (2)	0.0026 (2)	0.0057 (3)
C3	0.0192 (3)	0.0260 (4)	0.0202 (3)	0.0055 (3)	0.0071 (3)	0.0056 (3)
C4	0.0232 (3)	0.0210 (3)	0.0150 (3)	0.0042 (3)	0.0047 (3)	0.0022 (3)
C5	0.0169 (3)	0.0183 (3)	0.0134 (3)	−0.0001 (2)	0.0008 (2)	0.0002 (2)
C6	0.0119 (3)	0.0171 (3)	0.0132 (3)	0.0001 (2)	0.0016 (2)	0.0007 (2)
C7	0.0114 (2)	0.0171 (3)	0.0136 (3)	0.0003 (2)	0.0006 (2)	0.0003 (2)
C8	0.0116 (2)	0.0170 (3)	0.0116 (3)	−0.0001 (2)	0.0009 (2)	0.0005 (2)
C9	0.0124 (3)	0.0165 (3)	0.0119 (3)	−0.0003 (2)	0.0002 (2)	−0.0008 (2)
C10	0.0145 (3)	0.0229 (3)	0.0144 (3)	0.0006 (2)	0.0006 (2)	0.0010 (2)
C11	0.0137 (3)	0.0219 (3)	0.0125 (3)	−0.0006 (2)	−0.0003 (2)	0.0008 (2)
C12	0.0169 (3)	0.0237 (4)	0.0138 (3)	−0.0005 (3)	−0.0008 (2)	0.0042 (3)
C13	0.0115 (3)	0.0256 (4)	0.0186 (3)	0.0024 (2)	−0.0004 (2)	0.0030 (3)

### Geometric parameters (Å, °)

**Table:**

O1—N4	1.3789 (9)	C3—C4	1.3898 (13)
O1—C10	1.4108 (10)	C3—H3A	0.993 (14)
O2—C9	1.2340 (9)	C4—C5	1.3926 (11)
O3—C10	1.2205 (9)	C4—H4A	0.992 (15)
N1—N2	1.3933 (8)	C5—C6	1.3941 (10)
N1—C9	1.4030 (9)	C5—H5A	0.978 (13)
N1—C6	1.4252 (9)	C7—C8	1.3801 (10)
N2—C7	1.3450 (9)	C7—C13	1.4821 (10)
N2—C12	1.4626 (10)	C8—C9	1.4373 (10)
N3—N4	1.3169 (9)	C10—C11	1.4113 (10)
N3—C11	1.3494 (10)	C11—H11A	0.944 (13)
N3—C8	1.4060 (9)	C12—H12A	0.939 (16)
C1—C6	1.3896 (10)	C12—H12B	0.984 (13)
C1—C2	1.3916 (11)	C12—H12C	0.964 (15)
C1—H1A	0.981 (13)	C13—H13A	0.958 (15)
C2—C3	1.3920 (13)	C13—H13B	0.993 (16)
C2—H2A	0.962 (15)	C13—H13C	0.962 (16)
			
N4—O1—C10	110.85 (6)	C5—C6—N1	120.48 (6)
N2—N1—C9	109.54 (5)	N2—C7—C8	107.80 (6)
N2—N1—C6	119.34 (6)	N2—C7—C13	120.80 (7)
C9—N1—C6	124.51 (6)	C8—C7—C13	131.39 (7)
C7—N2—N1	109.25 (6)	C7—C8—N3	126.63 (6)
C7—N2—C12	125.52 (6)	C7—C8—C9	110.00 (6)
N1—N2—C12	120.54 (6)	N3—C8—C9	123.32 (6)
N4—N3—C11	115.11 (6)	O2—C9—N1	124.94 (6)
N4—N3—C8	118.67 (6)	O2—C9—C8	131.77 (7)
C11—N3—C8	126.22 (6)	N1—C9—C8	103.28 (6)
N3—N4—O1	104.05 (6)	O3—C10—O1	120.01 (7)
C6—C1—C2	118.78 (7)	O3—C10—C11	135.75 (7)
C6—C1—H1A	120.5 (8)	O1—C10—C11	104.24 (6)
C2—C1—H1A	120.7 (8)	N3—C11—C10	105.74 (6)
C1—C2—C3	120.87 (7)	N3—C11—H11A	122.8 (8)
C1—C2—H2A	120.5 (9)	C10—C11—H11A	131.5 (8)
C3—C2—H2A	118.6 (9)	N2—C12—H12A	108.2 (10)
C4—C3—C2	119.75 (7)	N2—C12—H12B	110.2 (8)
C4—C3—H3A	118.7 (8)	H12A—C12—H12B	107.1 (12)
C2—C3—H3A	121.6 (9)	N2—C12—H12C	111.3 (9)
C3—C4—C5	120.08 (8)	H12A—C12—H12C	112.3 (13)
C3—C4—H4A	119.2 (9)	H12B—C12—H12C	107.6 (12)
C5—C4—H4A	120.7 (9)	C7—C13—H13A	108.6 (9)
C4—C5—C6	119.46 (7)	C7—C13—H13B	107.8 (9)
C4—C5—H5A	119.9 (8)	H13A—C13—H13B	111.8 (12)
C6—C5—H5A	120.7 (8)	C7—C13—H13C	110.5 (9)
C1—C6—C5	121.04 (7)	H13A—C13—H13C	111.3 (13)
C1—C6—N1	118.48 (7)	H13B—C13—H13C	106.8 (13)
			
C9—N1—N2—C7	−3.85 (9)	N2—C7—C8—N3	176.29 (7)
C6—N1—N2—C7	−156.60 (7)	C13—C7—C8—N3	−5.07 (14)
C9—N1—N2—C12	−160.86 (7)	N2—C7—C8—C9	−1.23 (9)
C6—N1—N2—C12	46.38 (10)	C13—C7—C8—C9	177.42 (8)
C11—N3—N4—O1	−1.06 (9)	N4—N3—C8—C7	−24.91 (12)
C8—N3—N4—O1	179.40 (6)	C11—N3—C8—C7	155.61 (8)
C10—O1—N4—N3	1.21 (9)	N4—N3—C8—C9	152.29 (8)
C6—C1—C2—C3	−0.73 (12)	C11—N3—C8—C9	−27.19 (12)
C1—C2—C3—C4	0.93 (13)	N2—N1—C9—O2	−176.15 (7)
C2—C3—C4—C5	−0.10 (13)	C6—N1—C9—O2	−25.12 (12)
C3—C4—C5—C6	−0.92 (12)	N2—N1—C9—C8	2.90 (8)
C2—C1—C6—C5	−0.31 (12)	C6—N1—C9—C8	153.93 (7)
C2—C1—C6—N1	−179.62 (7)	C7—C8—C9—O2	177.90 (8)
C4—C5—C6—C1	1.14 (12)	N3—C8—C9—O2	0.29 (13)
C4—C5—C6—N1	−179.58 (7)	C7—C8—C9—N1	−1.05 (8)
N2—N1—C6—C1	−152.08 (7)	N3—C8—C9—N1	−178.67 (7)
C9—N1—C6—C1	59.49 (10)	N4—O1—C10—O3	178.89 (8)
N2—N1—C6—C5	28.61 (11)	N4—O1—C10—C11	−0.94 (9)
C9—N1—C6—C5	−119.82 (8)	N4—N3—C11—C10	0.50 (10)
N1—N2—C7—C8	3.07 (9)	C8—N3—C11—C10	180.00 (7)
C12—N2—C7—C8	158.67 (7)	O3—C10—C11—N3	−179.50 (10)
N1—N2—C7—C13	−175.74 (7)	O1—C10—C11—N3	0.29 (9)
C12—N2—C7—C13	−20.15 (12)		

### Hydrogen-bond geometry (Å, °)

**Table:**

*D*—H···*A*	*D*—H	H···*A*	*D*···*A*	*D*—H···*A*
C1—H1A···O3^i^	0.981 (14)	2.492 (13)	3.2155 (10)	130.4 (10)
C11—H11A···O2^i^	0.944 (13)	2.543 (13)	3.4163 (10)	153.9 (11)
C12—H12B···O2^ii^	0.984 (13)	2.369 (13)	3.3022 (10)	158.1 (11)
C13—H13A···O3^iii^	0.958 (14)	2.516 (15)	3.3606 (10)	147.0 (12)

Symmetry codes: (i) −*x*+1, −*y*+2, −*z*; (ii) −*x*+1, *y*−1/2, −*z*+1/2; (iii) −*x*+2, −*y*+2, −*z*.
